# Identification of a highly efficient stationary phase promoter in *Bacillus subtilis*

**DOI:** 10.1038/srep18405

**Published:** 2015-12-17

**Authors:** Xiaoxia Yu, Jiangtao Xu, Xiaoqing Liu, Xiaoyu Chu, Ping Wang, Jian Tian, Ningfeng Wu, Yunliu Fan

**Affiliations:** 1 Biotechnology Research Institute, Chinese Academy of Agricultural Sciences, Beijing 100081, China

## Abstract

A promoter that enabled high-level expression of the target gene during the stationary phase in the absence of an inducer would facilitate the efficient production of heterogeneous proteins at a low cost. In this study, a genome-scale microarray-based approach was employed to identify promoters that induced high-level expression of the target genes in *Bacillus subtilis* from the late log phase to the stationary phase without an inducer. Eleven candidate promoters were selected based on *B. subtilis* microarray data and the quantitative PCR analysis. Among the selected promoters, P*ylb* exhibited the highest activity with the reporter *bgaB* during the stationary phase. Compared with P43 (a commonly used constitutive promoter), promoter P*ylb* could express two reporter genes (*egfp* and *mApple*), and the expression levels of EGFP and RFP were 7.8- and 11.3-fold higher than that of P43, respectively. This finding was verified by overexpression of the genes encoding pullulanase and organophosphorus hydrolase, the activities of which were 7.4- and 2.3-fold higher, respectively, when driven by P*ylb* compared with P43. Therefore, our results suggest that the P*ylb* promoter could be used to overexpress target genes without an inducer; this method could facilitate the identification and evaluation of attractive promoters in the genome.

As a Gram-positive bacterium, *Bacillus subtilis* is an attractive host for the production of heterologous secretory proteins. *B. subtilis* is a nonpathogenic bacterium that can efficiently secrete a target protein into the culture medium. Much information concerning large-scale fermentation and production technology using this bacterium is available^1^,^2^,^3^. In addition, the genome and transcriptome in different conditions of *B. subtilis* have been determined^4^,^5^,^6^.

Promoters are important genomic regulatory elements that directly affect gene expression levels. In bacteria, RNA polymerases and associated sigma factors recognize promoters and are recruited by the binding of regulatory proteins to specific sites within promoters. To date, three types of promoter have been used for the high-level expression of heterologous proteins in *B. subtilis*: constitutive promoters, inducer-specific promoters, and auto-inducible promoters^1^,^7^,^8^,^9^,^10^. Although inducer-specific promoters (e.g., P_*spac*_ and P_*xyl*_) are the most widely used type, the requirement for inducer compounds—such as IPTG and xylose—could increase the cost of their industrial application^11^,^12^,^13^,^14^,^15^,^16^. Constitutive promoters, such as P43, are not suitable for production of toxic proteins. Auto-inducible promoters are ideal for large-scale protein production. Such promoters induce expression of the target gene from the late log phase to the stationary phase with no requirement for an inducer, which facilitates efficient production of heterogeneous proteins at a low cost. However, low activity^1^,^17^ is a barrier to the widespread use of the auto-inducible promoters available at present. Therefore, there is a need to identify novel auto-inducible promoters with high activity.

Here, we described a genome-wide microarray-based approach to identifying auto-inducible promoters in *B. subtilis* based on the report by Evert-Jan *et al.*^18^. A total of 58 stable phase-preferred and highly expressed genes were identified by microarray analysis. Among these genes (some of which were multicistronic), 21 stably phase-overexpressed genes with expression levels higher than that of *cdd* under the control of the P43 promoter (a commonly used constitutive promoter)^3^,^19^,^20^ were further evaluated by quantitative reverse transcriptase-PCR. Among the selected promoters, promoter P*ylb* was the most potential promoter. Circularized RNA reverse-transcription PCR indicated that the transcription initiation site of the P*ylb* promoter was an adenine, and the key elements (−10 box and −35 box) of the promoter were determined by site-directed mutagenesis. These results indicated P*ylb* to be an attractive and highly active promoter.

## Results

### SAM analysis and real-time PCR

To identify the target promoter, *B. subtilis* genome-wide microarray data were downloaded from NCBI (Accession No. GSE19831) and analyzed using the SAM software (Figure S5). The microarray data covered a total of 4,169 *B. subtilis* genes and 40 time points. The time points (1–4, 5–17, 19–26, and 27–40) represented the ‘lag’, log, early stationary, and late stationary growth phases, respectively. As shown in Figure S5A, a number of genes were highly expressed during the early and (or) late stationary growth phase. The expression profiles of those genes were shown in Figure S5B. A total of 58 genes were highly expressed during early and (or) late stationary growth phase, and their expression levels were higher than that of *cdd* gene which controlled by P43 (indicated by an arrowhead). The P43 promoter was selected as the positive control as it was commonly used strong promoter for *B. subtilis*^21^,^22^,^23^,^24^.

qRT-PCR was used to verify the expression levels of the 58 genes. RNA was extracted after 6, 12, 18, and 24 h (Figure S1). The first two time points (6 and 12 h) represented the log phase, and the later two (18 and 24 h) represented the stationary growth phase. The 16S rRNA gene was used as the reference gene, and the *cdd* gene under the control of the P43 promoter was used as a positive control. The results showed that the selected 21 genes could be divided into three categories according to their expression level (Figure S6). The transcript levels of genes *argD, yceC, yuaF, msmX, pbpE, rapA, sigW, yvlA, yxbB, ylbP, yobJ, yqeZ* and *yddF* were higher than that of *cdd* (Figure S6B,C), whereas the transcript levels of the other 8 genes were lower than that of *cdd* (Figure S6A). Among the selected genes, the transcript levels of *rapA* and *yddF* were highest (Figure S6C). However, their transcript levels were unstable during the stationary phase. In addition, *ylbP* was an attractive gene, as it exhibited very high activity during the stationary phase of growth but low activity in log phase. Therefore, we focused on the P*ylb* promoter which controlled *ylbP* in this study.

### Evaluation of the selected promoters from *B. subtilis*

**Figure 1:**
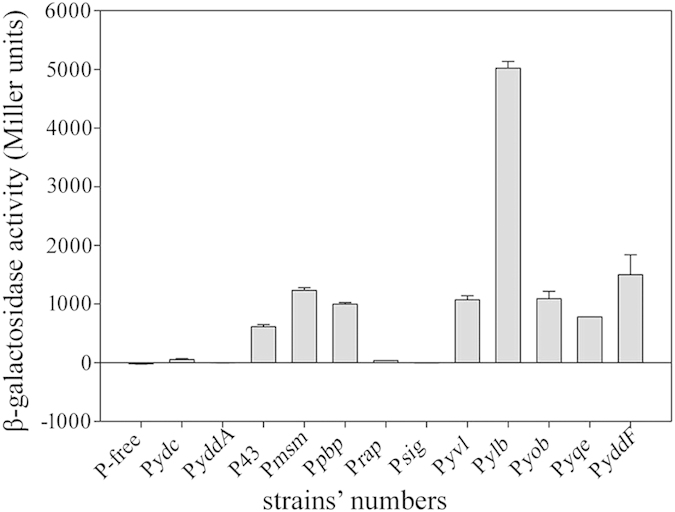
β-Galactosidase activities of the eleven candidate promoter clones in *B.* *subtilis*. β-Galactosidase production by the eleven candidate promoter clones in *B. subtilis* WB600 after 18 h of culture. Data are averages of three independent experiments. P-free: *B. subtilis* WB600 containing the P-free-bgaB-pUBC19 plasmid as a negative control. P43: *B. subtilis* WB600 containing the P43-bgaB-pUBC19 plasmid as a positive control. P*ydc*, P*yddA*, P*msm*, P*pbp*, P*rap*, P*sig*, P*yvl*, P*ylb*, P*yob*, P*yqe* and P*yddF*: *B. subtilis* WB600 strains containing the P*ydc*-bgaB-pUBC19, P*yddA*-bgaB-pUBC19, P*msm*-bgaB-pUBC19, P*pbp*-bgaB-pUBC19, P*rap*-bgaB-pUBC19, P*sig*-bgaB-pUBC19, P*yvl*-bgaB-pUBC19, P*ylb*-bgaB-pUBC19, P*yob*-bgaB-pUBC19, P*yqe*-bgaB-pUBC19 and P*yddF*-bgaB-pUBC19 plasmids, respectively.

Eleven candidate genes’ promoters (P*ydc*, P*yddA*, P*msm*, P*pbp*, P*rap*, P*sig*, P*yvl*, P*ylb*, P*yob*, P*yqe* and P*yddF*) were selected based on the results of SAM and qRT-PCR. Among the eleven promoters, the gene transcriptional levels driven by P*pbp*, P*rap*, P*sig*, P*ylb*, P*yqe* and P*yddF* were higher than that of P43, the gene transcriptional levels driven by P*msm*, P*yvl* and P*yob* were near to that of P43, and the gene transcriptional levels driven by P*ydc* and P*yddA* were lower than that of P43. To assess the activity of the eleven candidate promoters, 500–600 base pairs (bp)^25^ located upstream of the start codons were cloned from the genomic DNA of *B. subtilis* WB600 and inserted into the region upstream of the reporter gene *bgaB*. The promoter P43 was used as a positive control. The β-galactosidase activities of these eleven recombinants after culture for 18 h were shown in Fig. 1, and the results of the SDS-PAGE analysis of BgaB in crude extract were shown in Figure S7. Among the selected promoters, the β-galactosidase activity driven by P*ylb* was highest, 8.2-fold higher than that of P43. However, the β-galactosidase activity driven by P*yddF* was only 2.4-fold higher than that driven by P43. This may be because P*yddF* is a log-phase-specific promoter; Indeed, it resulted in the highest β-galactosidase activity in a log phase, 7.2-fold higher than that driven by P43 (Figure S8). Moreover, the β-galactosidase activities driven by P*msm*, P*pbp*, P*yvl*, P*yob* and P*yqe* were ~2.0, 1.6, 1.7, 1.8 and 1.3-fold, respectively, of those driven by P43. In contrast, the P*ydc* and P*yddA* promoters resulted in negligible β-galactosidase activity. However, the clones of P*rap* and P*sig* promoters could be observed blue on the LB agar containing 10 μg/mL kanamycin with x-gal (Figure S9). But it was difficultly to detect the β-galactosidase activity on several different time points in liquid LB medium. All of the results indicated that the promoter P*ylb* was a good candidate promoter to express the target gene.

### P43 and P*ylb*-driven fluorescent protein expression *B. subtilis*

**Figure 2:**
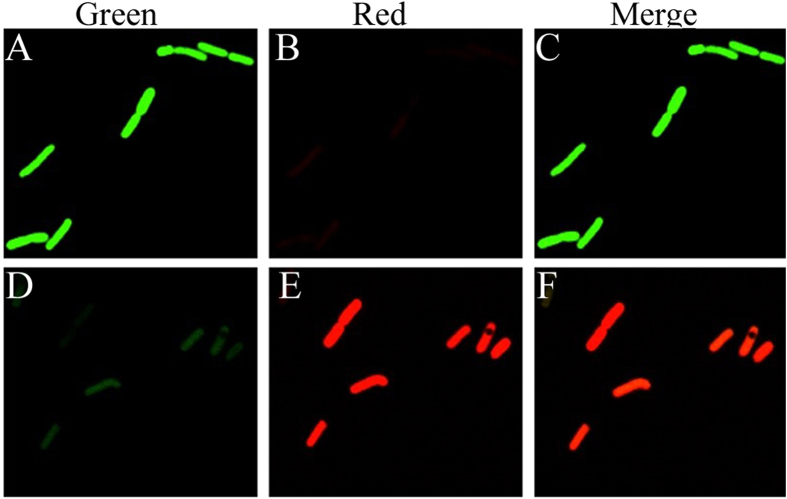
Fluorescence micrographs of *B. subtilis* WB600 harboring the P*ylb*-G-P43-R-pUBC19 and P*ylb*-R-P43-G-pUBC19 plasmids. (**A**) Green fluorescence micrograph, (**B**) Red fluorescence micrograph and (**C**) Merge of green fluorescence and red fluorescence of *B. subtilis* WB600 harboring the P*ylb*-G-P43-R**-**pUBC19 plasmid after 18 h of culture. (**D**) Green fluorescence micrograph, (**E**) Red fluorescence micrograph and (**F**) Merge of green fluorescence and red fluorescence of *B. subtilis* WB600 harboring the P*ylb*-R-P43-G-pUBC19 plasmid after 18 h of culture. Images were acquired using a Nikon microscope with a 100 × objective.

To compare the activity level of the P*ylb* promoter to that of P43, we used the green (EGFP) and red (mApple) fluorescent proteins as reporters. To prevent any influence of plasmid copy number and cell concentration on promoter activity, three fusion plasmids (G-R-pUBC19, P*ylb*-R-P43-G-pUBC19, and P*ylb*-G-P43-R-pUBC19), each of which contained two promoters and two reporter genes, were constructed and transformed into *B. subtilis* WB600. EGFP and mApple have similar fluorescence intensities; These proteins exhibit relative brightness levels of 100 and 109, respectively^26^. In the plasmid P*ylb*-R-P43-G-pUBC19, the P*ylb* and P43 promoters drove the *mApple* and *egfp* reporter genes, respectively. Green and red fluorescence were measured in the recombinant strain harboring the P*ylb*-R-P43-G-pUBC19 plasmid. If the activity of the P*ylb* promoter were greater than that of P43, the red fluorescence intensity would be greater than that of green fluorescence. The recombinant strain containing the plasmid E-R-pUBC19 was used as a negative control; no fluorescence was detected in this strain. As shown in Fig. 2, the strains containing the plasmids P*ylb*-G-P43-R-pUBC19 and P*ylb*-R-P43-G-pUBC19 exhibited green and red fluorescence after culture for 18 h. These results indicated that the activity of the P*ylb* promoter was greater than that of P43.

**Figure 3:**
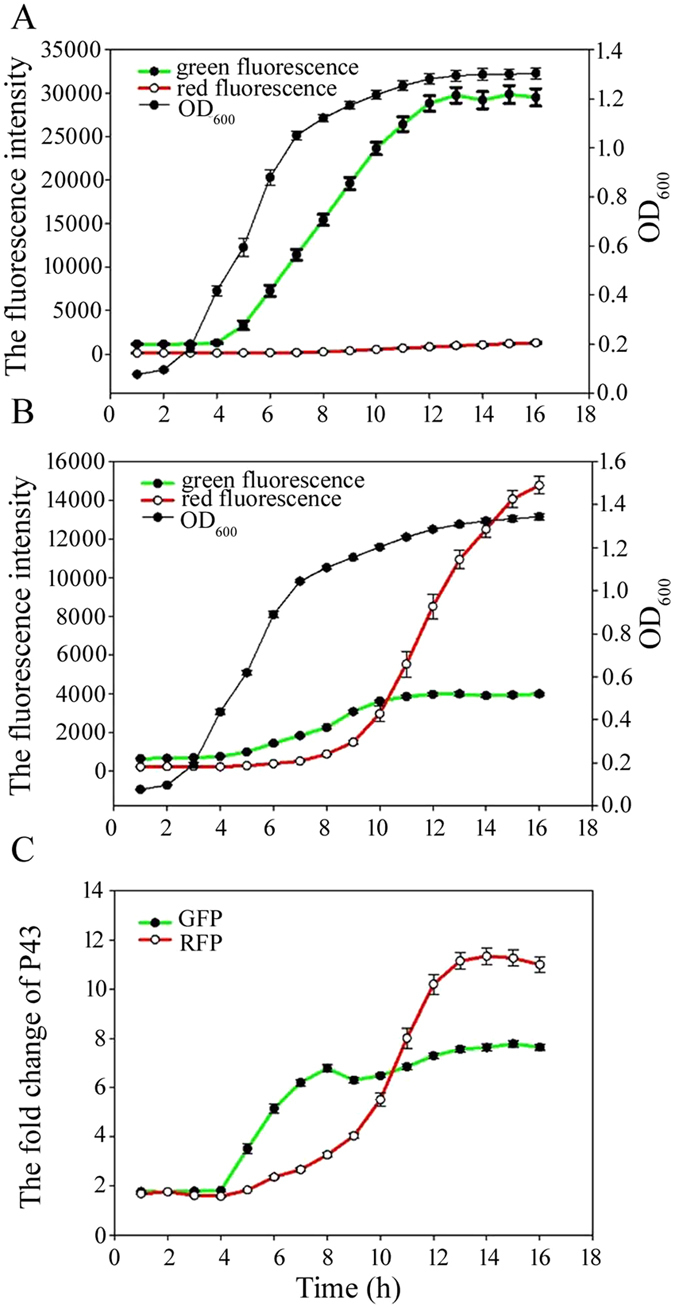
Fluorescence intensity and growth curves of *B. subtilis* harboring the P*ylb*-G-P43-R-pUBC19 and P*ylb*-R-P43-G-pUBC19 plasmids. (**A**) Fluorescence intensity and growth curve of *B. subtilis* WB600 harboring the P*ylb*-G-P43-R-pUBC19 plasmid. (**B**) Fluorescence intensity and growth curve of *B. subtilis* WB600 harboring the P*ylb*-R-P43-G-pUBC19 plasmid. (**C**) Fold-change in Flu/OD_600_ (fluorescence units per OD_600_) for P*ylb* relative to P43. *B. subtilis* WB600 containing the P*ylb*-G-P43-R-pUBC19 or P*ylb*-R-P43-G-pUBC19 fusion plasmid was grown in LB medium containing 10 μg/mL kanamycin. Data are averages of three independent experiments.

To quantitatively assess the activity of the promoters, the fluorescence intensity of strains containing the P*ylb*-G-P43-R-pUBC19 or P*ylb*-R-P43-G-pUBC19 plasmid was monitored spectrophotometrically every hour. The results of the strain containing the recombinant plasmid P*ylb*-G-P43-R-pUBC19 indicated that the green fluorescence intensity driven by P*ylb* was higher than the red fluorescence intensity driven by P43 (Fig. 3A), and the strain containing the recombinant plasmid P*ylb*-R-P43-G-pUBC19 showed that the red fluorescence intensity driven by P*ylb* was higher than the green fluorescence intensity driven by P43 (Fig. 3B). These results indicated that the P*ylb* promoter could induce high-level expression of GFP and RFP.

We also determined the time point at which the P*ylb* promoter was expressed and compared it with P43. The P*ylb* promoter resulted in EGFP and mApple reporter protein expression beginning during log and stationary phases, respectively (Fig. 3A,B). Therefore, the P*ylb* promoter is capable of inducing expression of its target gene from the log phase to the stationary phase. As the folding rates may vary among proteins^27^, the time at which a target gene is expressed may be different. The reporter protein expression ratio (EGFP and mApple) between the P*ylb* and P43 promoters at various time points was shown in Fig. 3C. The reporter protein (EGFP and mApple) expression level was similar during the lag and early log phases. However, in the late log and stationary phases, the reporter protein expression level driven by the P*ylb* promoter was significantly higher than that driven by P43. P*ylb*-induced EGFP and mApple levels of expression after 15 h of culture were 7.8 and 11.3-fold, respectively, higher than that of P43. These results indicated that the P*ylb* promoter was capable of inducing expression of the target protein from the late log phase to the stationary phase with no requirement for an inducer and that the P*ylb* promoter exhibited higher activity than P43.

### Determination of the transcription start site of the P*ylb* promoter

**Figure 4:**
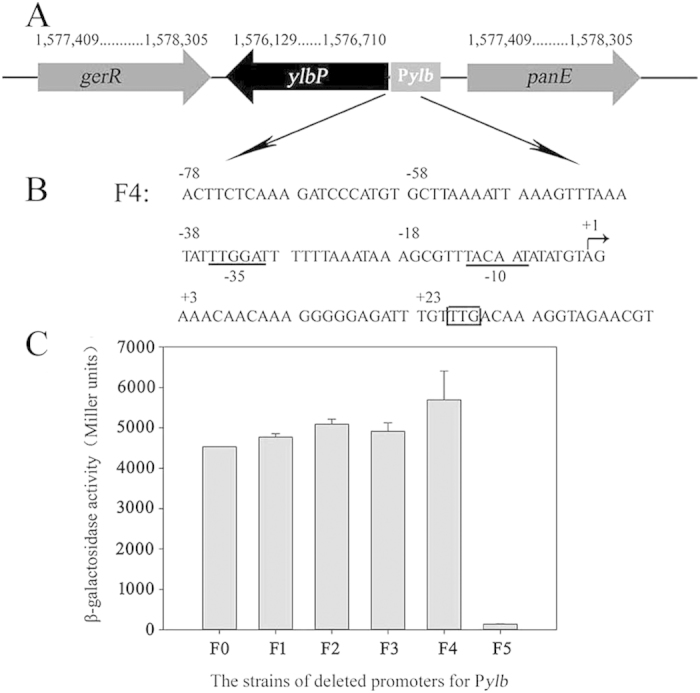
Map of the *ylbP* gene, and the promoter sequence and β-galactosidase activity of the deleted promoters clones in *B. subtilis*. (**A**) Map showing the *ylbP* gene in the *B. subtilis* genome, and *ylbP* encodes an N-acetyltransferase. (**B**) Sequence of the P*ylb* promoter. The start codon of *ylbP* is shown in the square. The arrow indicates the transcription start site of the P*ylb* promoter. The −10 box and −35 element are indicated by underlines. (**C**) The β-galactosidase activity of the *B. subtilis* strains in which the P*ylb* promoter was deleted. Data are averages of three independent experiments.

In the *B. subtilis* genome, the P*ylb* promoter controls the expression of *ylbP*, which is located at 1,576,129–1,576,710 on the complementary strand, upstream of *gerR* and downstream of *panE*. Our analyses revealed that the *ylbP* gene was monocistronic (Fig. 4A). The CR-RT-PCR method was used to determine the transcription start site of the P*ylb* promoter. RNA was extracted from *B. subtilis* WB600 containing the F0-bgaB-pUBC19, F3-bgaB-pUBC19, and F4-bgaB-pUBC19 fusion plasmids after 18 h of culture. Sequencing analysis revealed that the transcriptional start site was an adenine (Fig. 4B). To experimentally analyze the core element of the P*ylb* 5′-flanking region, analysis of the effect of P*ylb* deletion was performed using the *bgaB* fusion reporter system. The β-galactosidase activities of the resulting constructs were measured (Fig. 4C), and SDS-PAGE analysis of BgaB was performed (Figure S10). The *B. subtilis* strain harboring the wild-type P*ylb* promoter (F0) and those with deletions (F1, F2, F3 and F4) exhibited similar β-galactosidase activities. However, the *B. subtilis* strain containing the F5 fusion plasmid exhibited negligible β-galactosidase activity. These results revealed that the F4 strain contained the main core element, but F5 was deficient in the −35 element. Therefore, the putative recognition element (−35 TTGGAT −30) and a −10 box (−12 TACAAT −7) were presented in the P*ylb* promoter.

### Construction of mutants of promoter P*ylb* by site-directed mutagenesis

**Figure 5:**
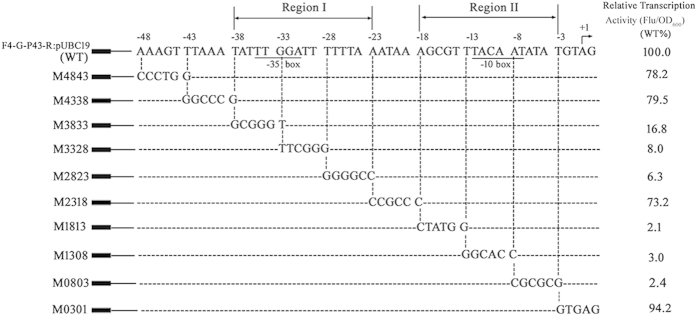
Site-directed mutagenesis from bp −48 to −1 of the P*ylb* promoter. The DNA sequence of wild-type P*ylb* is shown at the top (WT). The transcription start site (TSS) of P*ylb* was identified by CR-RT-PCR. The mutated nucleotides of the M4843 to M0301 mutants are shown below the WT promoter sequence. Green fluorescence intensity was determined using a microplate reader. The fold-changes in Flu/OD_600_ (fluorescence units per OD_600_) relative to the WT (set to 100%) indicate the transcriptional activities of the mutants. The significant fold-changes in strains M3833, M3328, M1308, and M0803 were due to large reductions in fluorescence intensity. Data are averages of three independent experiments, each of which comprised four replicates.

The above results demonstrated that the shortest functional sequence of P*ylb* was F4 and revealed its putative recognition element (−35 box and −10 box). To experimentally analyze the cis-acting elements of P*ylb* in detail, site-directed mutagenesis was performed using the EGFP fusion reporter system with the F4-egfp-pUC19 plasmid (see Materials and methods). Thus the promoter region from bp −48 to −1 of F4 was analyzed using site-directed scanning mutagenesis to pinpoint the regions that mediate the transcriptional activity of P*ylb*. The resulting constructs were named pM4843 to pM0301; the numbers indicated the mutated region. For example, pM4843 indicated mutagenesis from bp −48 to −43 relative to the transcription start site of P*ylb*. These plasmids were transformed into *B. subtilis* WB600 to generate the reporter strains M4843 to M0301, which exhibited green fluorescence (Fig. 5). Mutations in the putative −35 box (−35 TTGGAT −30) regions (M3833 and M3328) and −10 element (−12 TACAAT −7) regions (M1308 and M0803) led to complete loss of transcriptional activity. In addition, the region from bp −28 to −23 and that from bp −18 to −13 might be directly involved in the transcriptional activity of P*ylb*. Notably, the green fluorescence intensity did not significantly change the transcriptional levels of the mutants M4843, M4338, M2318, and M0301, indicating that the corresponding regions in these mutants were not directly involved in the transcriptional activity of P*ylb*. These results revealed that the promoter regions I (bp −38 to −23) and II (bp −18 to −3) were particularly important for the transcriptional activity of P*ylb*.

### The application prospect of the promoter P*ylb*

**Figure 6:**
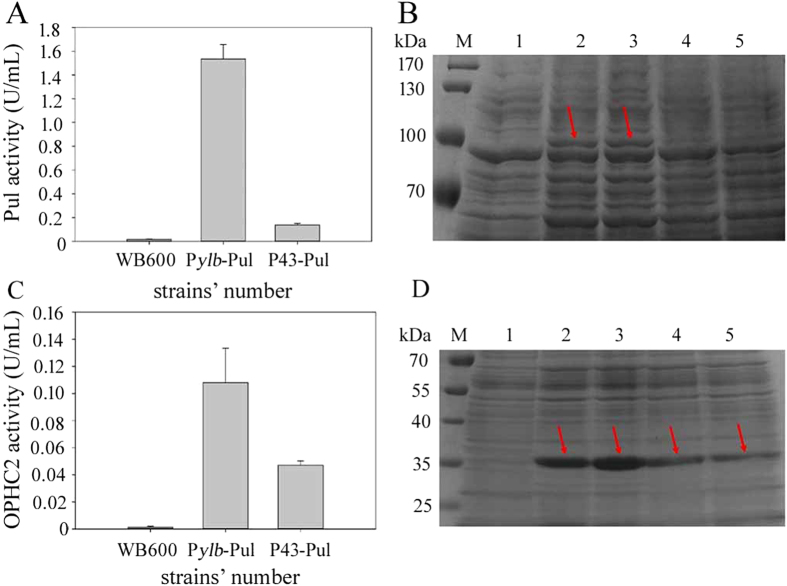
Pullulanse and OPHC2 activities of *B. subtilis* strains harboring various promoters after 18 h of culture. (**A**) Production of pullulanse by *B. subtilis* WB600 harboring the recombinant plasmid P*ylb*-ZDs-Pul-pUBC19 (indicated by P*ylb*-Pul), P43-ZDs-Pul-pUBC19 (P43-Pul), or no recombinant plasmid (WB600) after 18 h of culture. (**B**) SDS-PAGE analysis of pullulanse activity in the culture supernatant that was concentrated about 15 folds after 18 h of culture. M: protein molecular mass marker. 1: *B. subtilis* WB600 as a negative control. 2 and 3: P*ylb*-Pul-1, P*ylb*-Pul-2. 4 and 5: P43-Pul-1 and P43-Pul-2, and the arrows indicate target protein pullulanse. (**C**) Production of OPHC2 by *B. subtilis* WB600 harboring the recombinant plasmid P*ylb*-ZDs-OPHC2-pUBC19 (indicated by P*ylb*-OPHC2), P43-ZDs-OPHC2-pUBC19 (P43- OPHC2), and no recombinant plasmid (WB600) after 18 h of culture. (**D**) SDS-PAGE analysis of OPHC2 in the culture supernatant that was concentrated about 15 folds after 18 h of culture. M: protein molecular mass marker. 1: *B. subtilis* WB600 as a negative control. 2 and 3: P*ylb*-OPHC2-1, P*ylb*-OPHC2-2. 4 and 5: P43-OPHC2-1 and P43-OPHC2-2, and the arrows indicate the target protein OPHC2. Data are averages of three independent experiments.

To evaluate the P*ylb* promoter’s potential for application, P*ylb*-ZDs/P43-ZDs (containing the promoter and signal peptide of amylase from *Bacillus amyloliquefaciens*) and two foreign genes, *pul*/*ophc2*, were cloned into the shuttle vector pUBC19. The Pul activities of the P*ylb*-ZDs-Pul-pUBC19 and P43-ZDs-Pul-pUBC19 recombinants after 18 h of culture were shown in Fig. 6A, and the OPHC2 activities were presented in Fig. 6C. Our results confirmed that the P*ylb* promoter induced significantly higher levels of expression of Pul and OPHC2 than did P43. The P*ylb*-driven Pul activity was 7.4-fold higher than that driven by P43, and the P*ylb*-driven OPHC2 activity was 2.3-fold higher than that driven by P43. The results of SDS-PAGE analysis of Pul and OPHC2 in the culture supernatant after concentrated about 15 folds were shown in Fig. 6B,D, respectively. Moreover, high levels of expression of heterologous proteins did not significantly affect the growth of the recombinant *B. subtilis* WB600 strains (data not shown). In addition, cultivations were also performed in SB medium, as shown the supplementary material. The results of Pul and OPHC2 activity and the SDS-PAGE analysis were shown in Figure S11. It also indicated that the activity of P*ylb* promoter was stronger than that of P43 when driving Pul and OPHC2. Thus, the P*ylb* promoter may have potential for the overexpression of proteins in *B. subtilis*.

## Discussion

One of the key factors for achieving high-level expression of heterologous genes is the use of a strong and controllable promoter. Despite the fact that several strong promoters have been reported^7^,^10^,^28^,^29^,^30^,^31^,^32^, industrial production using *B. subtilis* is usually mediated by inducer-specific promoters. In comparison, auto-inducible promoters have a distinct advantage in terms of not requiring an inducer compound, which simplifies the industrial production of the target protein and reduces the cost. Thus, identification of potential auto-inducible promoters would be beneficial.

Nowadays, much information about transcriptome data of many species is available on line, which will help to identify the useful promoters^6^,^18^. However, in addition to the strength of the promoter, there are many variables affecting expression levels, including the stability of the mRNA, the culture conditions, growth situation of the cell and so on. Therefore, for the aim to isolate the useful promoter, we need not only analyze the transcriptome date, but also use the reporter genes and systematically study the function of the promoter.

Our results indicated that the P*ylb* promoter induced sufficiently high levels of expression in *B. subtilis* of active β-galactosidase, EGFP, RFP, pullulanase, and organophosphorus hydrolase from the late log phase to the stationary phase. In addition, the production of those foreign proteins was higher than that induced by the P43 promoter, which has been used widely in *B. subtilis*. Moreover, the high expression level of heterologous proteins did not significantly affect the growth of *B. subtilis*. Thus, the P*ylb* promoter may have potential for the overexpression of useful proteins in *B. subtilis*.

According to the *B. subtilis* genome sequence, the P*ylb* promoter controls the *ylbP* gene, which has a length of 483 bp and encodes an N-acetyltransferase. The gene could be expressed in the stationary stage, which has also been confirmed by the transcriptome study in the different conditions, as shown in Subwiki^6^. This enzyme encoded by *ylbP* is capable of catalyzing the transfer of acetyl groups between acetyl coenzyme A and amines. Thus, it may be involved in post-translational modification in *B. subtilis* during the stationary phase. However, the function of and the reason that the *ylbP* gene was highly expressed from the late log phase to the stationary phase remain unclear.

This study is an example of the discovery of an interesting promoter using a genome-scale microarray-based approach. Genomic and gene expression profile data contain a large quantity of biological information, which could be exploited in various fields. Compared with the traditional promoter trap method^32^, the technique described herein is more effective in terms of identifying attractive promoters and will used to this end in future studies.

## Materials and Methods

### Bacterial strains, plasmids and growth conditions

*B. subtilis* WB600 and *Bacillus amyloliquefaciens* were stored in our laboratory. *Escherichia coli* Trans1-T1 was used as the host strain for the cloning experiment (Transgen Biotech, Beijing). The plasmids used in this study are listed in Table S1 in the supplemental material. The bacterial strains were cultured in Luria–Bertani (LB) medium at 37 °C. The following concentrations of antibiotics were used for selection: 100 μg/mL ampicillin (Amp), 10 μg/mL kanamycin (Kana), and 5 μg/mL tetracycline (Tet).

### Analysis of *B. subtilis* DNA microarray data

The *B. subtilis* microarray data (NCBI Accession No. GSE19831) were reported by Evert-Jan *et al.*^18^. *B. subtilis* exhibits four distinct growth phases: the lag phase, the log growth phase, and the early and late stationary growth phases. The microarray data were grouped into four growth phases, and the Significant Analysis of Microarray software (SAM; Stanford University) was employed to identify genes specifically expressed from the late log growth phase to the stationary phase.

### RNA extraction and qRT-PCR

*B. subtilis* WB600 was cultured at 37 °C overnight in LB medium. Then, a 1:100 volume of a fresh overnight *B. subtilis* culture was inoculated into 50 mL LB medium in a 100 mL flask and cultured at 37 °C with shaking at 200 rpm. Growth was monitored by measurement of the optical density at 550 nm (OD_550_) (Figure S1). *B. subtilis* cells were collected after 6, 12, 18, and 24 h of incubation and subjected to RNA extraction using an RNA Prep Pure Cell/Bacteria Kit (Tiangen Biotech, Beijing) according to the manufacturer’s instructions. To remove DNA contamination, 1 μg of diluted RNA was digested with DNase I (New England Biolabs, USA). Specific primers for the target DNA regions (Table S2) and suitable concentrations of the cDNA templates were used for qRT-PCR. Details of the qRT-PCR method are provided in the Materials and methods section of the supplementary material.

### Construction of a promoter trap vector with *bgaB* as the reporter gene

All primers used in this study are listed in Table S2. First, a promoter trap vector, P-free-bgaB-pUBC19, was constructed as described in the supplementary material. The selected alternative promoters were amplified from the genomic DNA of *B. subtilis* WB600 using the respective primer pairs, and the commonly used strong constitutive promoter P43 was amplified as the control promoter. The amplified promoters were digested with *Pst* I and *Xho* I and inserted into the plasmid P-free-bgaB-pUBC19, which had been treated with the same restriction endonucleases, and then transformed into *E. coli* strain Trans1-T1 to construct the corresponding fusion expression plasmid Promoter-bgaB-pUBC19. The fusion plasmid Promoter-bgaB-pUBC19 was constructed by means of the experimental procedure shown in Figure S2.

### Construction of binary expression vectors

Two termination sequences were added at the ends of the *egfp* (GenBank Accession No. JQ627826.1) and *mApple* (GenBank Accession No. HM771700.1) genes, and one termination sequence was added between the two genes. Moreover, the direction of the two genes was reversed. Multiple cloning sites were then added to the ends of *egfp* and *mApple* (Figure S3 in the supplemental material). After codon optimization based on the *B. subtilis* genome, the *egfp-mApple* gene was synthesized by Genscript Corporation (Nanjing, China). The synthetic *egfp-mApple* gene was located on the pUC57-Simple plasmid. The fusion egfp-mApple-pUC57 Simple plasmid was digested with *Hind* III and *BamH* I and inserted into the plasmid pUBC19, which had been treated with the same restriction endonucleases, and then transformed into *E. coli* Trans1-T1 to construct the corresponding fusion plasmid (G-R-pUBC19). Next, the promoters P43 and P*ylb* were inserted upstream of the *egfp* and *mApple*, respectively, and then transformed into *E. coli* Trans1-T1 to construct the corresponding fusion plasmids (P*ylb*-R-P43-G-pUBC19). Moreover, the promoters P43 and P*ylb* were inserted prior to *mApple* and *egfp*, respectively, and then transformed into *E. coli* Trans1-T1 to construct the corresponding fusion plasmids (P*ylb*-G-P43-R-pUBC19, see Figure S3 in the supplemental material).

### Construction of fusion plasmids for the truncated 5′-flanking sequence of the P*ylb* promoter

To analyze its characteristics in detail, five truncated fragments of the P*ylb* promoter (named F0) promoter −381– + 43 (F1), −233– + 43 (F2), −154– + 43 (F3), −78– + 43 (F4), and −21– + 43 (F5)—were amplified using the cognate primer pairs F1-up, F2-up, F3-up, F4-up, F5-up and P*ylb*-down (see Table S2 in the supplemental material). The PCR products of the five fragments were digested with *Pst* I and *Xho* I and inserted into the P-free-bgaB-pUBC19 plasmid to produce the corresponding fusion plasmids.

### Construction of scanning site-directed plasmids harboring the P*ylb* promoter

To analyze the regulatory region of the P*ylb* promoter, scanning site-directed mutants were constructed. Briefly, the fragment F4-egfp (containing the −78– + 43 fragment of the P*ylb* promoter and *egfp*) was amplified from the plasmid P*ylb*-G-P43-R-pUBC19 using the primers F4-egfp-up and F4-egfp-down. The amplified product F4-egfp was digested with *Xba* I and *Hind* III and inserted into the plasmid pUC19, which had been treated with the same restriction endonucleases, to construct the fusion plasmid F4-egfp-pUC19. Based on the plasmid F4-egfp-pUC19, 10 scanning site-directed mutational plasmids of the F4 fragment were constructed using a previously described two-step PCR method^33^. After confirmation by sequencing, the 10 scanning site-directed mutational F4-egfp and wild-type F4-egfp fragments were cleaved with *Xba* I and *Hind* III and inserted into the plasmid P*ylb*-G-P43-R-pUBC19, which had been treated with the same restriction endonucleases, to construct mutational plasmids (pM4843 to pM0301).

### Construction of plasmids for gene overexpression

The pullulanase and organophosphorus hydrolase (*ophc2*) genes were selected to evaluate the P*ylb* promoter’s potential for application in *B. subtilis*. The signal peptide sequence (ZDs) of α-amylase from *B. amyloliquefaciens* was inserted into the region upstream of the target genes. Four recombinant plasmids were constructed; the fusion plasmids P*ylb*-ZDs-Pul-pUBC19 and P43-ZDs-Pul-pUBC19 contained the F4 and P43 promoters, respectively, together with the pullulanase gene from *Bacillus naganoensis*. The other two fusion plasmids, P*ylb*-ZDs-OPHC2-pUBC19 and P43-ZDs-OPHC2-pUBC19, contained the organophosphorus hydrolase gene (*ophc2*) from *Pseudomonas pseudoalcaligenes*. The details of fusion plasmid construction are provided in the supplemental material (Figure S4).

### Transformation of plasmids into *B. subtilis* WB600

After confirmation by sequencing, the plasmids were extracted from *E. coli* Trans1-T1 and transformed into *B. subtilis* WB600 as described previously^34^. Transformants were harvested by screening the clones on LB agar containing 10 μg/mL kanamycin.

### Circularized RNA reverse-transcription-PCR

Circularized RNA reverse transcription PCR (CR-RT-PCR) was used to identify the transcription start site of the P*ylb* promoter. Extraction and circularization of RNA from *B. subtilis* WB600 harboring the F0-bgaB-pUBC19, F3-bgaB-pUBC19, and F4-bgaB-pUBC19 fusion plasmids were carried out as described previously^35^,^36^. Self-ligated RNA was reverse transcribed using the specific reverse primer PB1 (Table S2 in the supplemental material). The cDNA was first amplified using the specific primers PB2-up and PB2-down. Thereafter, to enhance the specificity, a second PCR was performed using the PB3-up and PB3-down inner primers. The purified products of the second PCR were cloned into the TA vector pGM-T (TianGen Biotech, Beijing) according to standard procedures, and 20 clones were subjected to sequencing.

### Determination of enzyme activity

The methods for determination of pullulanase, organophosphorus hydrolase, and β-galactosidase activities are described in the supplementary material.

### Fluorescence assay

*B. subtilis* WB600 cells containing the *eGFP* or *mApple* reporter gene were cultured for ~18 h, washed three times, and resuspended in 0.9% NaCl. A 5 μL volume of cells at an appropriate dilution in 0.9% NaCl was placed on a microscope slide and covered with a coverslip. Fluorescence microscopy was performed using a 100 × oil immersion lens (Nikon, Japan) to visualize cell fluorescence. To measure the fluorescence intensity, the constructed *B. subtilis* WB600 strains harboring the P*ylb*-R-P43-G-pUBC19 and P*ylb*-G-P43-R-pUBC19 plasmids were cultured in black, 96-well plates with a clear, flat bottom (Corning, USA) at 37 °C with shaking at 750 rpm in incubator 1000 (Heidolph, Germany). The fluorescence intensity was measured at 1-h intervals using a SpectraMax M2 instrument (Molecular Devices, USA). The excitation and emission wavelengths were 484 and 507 nm and 586 and 592 nm, for enhanced green fluorescent protein (EGFP)^26^ and red fluorescent protein (RFP)^37^, respectively. The values shown are the averages of three independent experiments.

## Additional Information

**How to cite this article**: Yu, X. *et al.* Identification of a highly efficient stationary phase promoter in *Bacillus subtilis. Sci. Rep.* **5**, 18405; doi: 10.1038/srep18405 (2015).

## Supplementary Material

Supplementary Information
