# Supplementary material for: Identification of a highly efficient stationary phase promoter in Bacillus subtilis
Source: Sci Rep. 2015 Dec 17;5:18405. doi: 10.1038/srep18405 (PMC4682092; doi:10.1038/srep18405)
Supplement: Supplementary Information [file srep18405-s1.pdf]

Identification of a highly efficient stationary phase promoter in *Bacillus subtilis*

Xiaoxia Yu, Jiangtao Xu, Xiaoqing Liu, Xiaoyu Chu, Ping Wang, Jian Tian*, Ningfeng Wu*, Yunliu Fan

Biotechnology Research Institute, Chinese Academy of Agricultural Sciences, Beijing 100081, China

*Corresponding author:

Jian Tian, Tel.: 86-10-82109864; Fax: 86-10-82109844; E-mail: tianjian@caas.cn

Ningfeng Wu, Tel.: 86-10-82109864. Fax: 86-10-82109844. E-mail: wuningfeng@caas.cn

Supplementary Materials and Methods

RNA extraction and qRT-PCR

The amplification and detection of target regions were performed on a real-time cycler (Bio-Rad iQ5, USA) under a standard three-step PCR procedure: initial denaturation at 95°C for 5 min, followed by 40 cycles of denaturation at 95°C for 15 sec, annealing at 60°C for 20 sec, and synthesis 72°C for 20 sec; a melting curve was generated by linear heating from 55°C to 95°C over 81 cycles. For cDNA synthesis, 500 ng of DNase I-treated total RNA were transcribed using a mixture of specific reverse primers with the TransScript One-Step gDNA Removal and cDNA Synthesis SuperMix (Transgen, Beijing). DNase I-treated RNA was used to check for genomic DNA contamination.

Construction of a promoter trap vector

To screen for and identify highly efficient promoters, a promoter trap vector was constructed. Briefly, the *bgaB* reporter gene was amplified from the pDK plasmid [1] using the primer pair bgaB-up and bgaB-down, which carried additional *Pst* I and *Xho* I sites at the 5'-end and *Bam*H I at 3'-end. The PCR products were digested and ligated with the shuttle plasmid pUBC19 at the 5'-end *Pst* I and 3'-end *Bam*H I restriction sites and then transformed into the *E. coli* Trans1-T1 strain to construct the promoter trap vector P-free-bgaB- pUBC19, which was used as a negative control plasmid.

Construction of plasmids for gene overexpression

To further exploit the application of the P*ylb* promoter in *B. subtilis*, four recombinant plasmids were constructed. Briefly, the P*ylb* promoter was amplified from the plasmid P*ylb*-bgaB-pUBC19 using F3-up and P*ylb*-down primers, and the promoter and signal peptide of amylase ZDsylb was amplified from the native plasmid of *B. amyloliquefaciens* using the primers ZDsylb -up and ZDs-down. The P*ylb* promoter and the signal peptide sequence of the amylase P*ylb*-ZDs were then amplified by overlapping-extension PCR using F3-up/ZDs-down as the primers and the PCR products of the P*ylb* promoter and ZDsylb as the template. The amplified product P*ylb*-ZDs was digested with *Xba* I and *Xho* I and stored. At the same time, the pullulanase gene (*pul*) from *Bacillus*

naganoensis was amplified by PCR using the primers Pul-up and Pul-down and the plasmid Pul-pET22b as the template. The amplified products were digested with *Xho* I and *Pst* I and stored. Finally, the amplified P*ylb*-ZDs fragment and the amplified *pul* fragment—which had been digested with the aforementioned two restriction enzymes—were inserted into the plasmid pUBC19, which had been treated with *Xba* I and *Pst* I, and then transformed into *E. coli* Trans1-T1 to construct the corresponding fusion plasmid P*ylb*-ZDs-Pul-pUBC19. The method used to construct the fusion plasmid P43-ZDs-Pul-pUBC19 was similar to that for P*ylb*-ZDs-Pul-pUBC19, with the exception that the P*ylb*-ZDs fragment was replaced by P43-ZDs, which was amplified by overlapping-extension PCR using P43-up and ZDs-down as the primers and the PCR products of the P43 promoter and ZDs43 as the template. The fusion plasmids P*ylb*-ZDs-Pul-pUBC19 and P43-ZDs-Pul-pUBC19 were constructed by means of the experimental procedure shown in Figure S4.

Based on the fusion plasmids P*ylb*-ZDs-Pul-pUBC19 and P43-ZDs-Pul-pUBC19, the organophosphorus hydrolase gene (*ophc2*) from *Pseudomonas pseudoalcaligenes* was amplified by PCR using the primers OPHC2-up/OPHC2-down and the plasmid OPHC2-pET30a as the template. The amplified products were digested with *Xho* I and *Pst* I and inserted into the plasmids P*ylb*-ZDs-Pul-pUBC19 and P43-ZDs-Pul-pUBC19, respectively, which had been treated with the same restriction endonucleases. The plasmids were then transformed into *E. coli* Trans1-T1 to construct the corresponding fusion plasmids P*ylb*-ZDs-OPHC2-pUBC19 and P43-ZDs-OPHC2-pUBC19.

β-Galactosidase activity assay

B. subtilis WB600 containing each fusion plasmid with the *bgaB* reporter gene was cultured at 37°C in an orbital shaker at 200 rpm in LB medium containing 10 μg/mL kanamycin. After incubation for 18 h, samples were taken for determination of β-galactosidase activities, as described previously [2]. The β-galactosidase specific activities were converted to Miller units, as described previously [3]. The values shown are the averages of three independent experiments.

Pullulanase and organophosphorus hydrolase activity assays

B. subtilis WB600 harboring the P*ylb*-ZDs-Pul-pUBC19 or P43-ZDs-Pul-pUBC19 (P*ylb*-ZDs-OPHC2-pUBC19 or P43-ZDs-OPHC2-pUBC19) plasmid was grown at 37°C in an orbital shaker

at 200 rpm in LB medium containing 10 μg/mL kanamycin, and *B. subtilis* WB600 containing no plasmid was grown under identical conditions, with the exception that the medium contained 5 μg/mL tetracycline. After culturing for 18 h, samples were taken for determination of pullulanase activities. Pullanase activity was assayed by quantifying the release of aldehyde groups in a mixture of pullulan solution and the diluted enzyme sample [4,5]. The reaction mixture, containing 500 μL 0.5% (w/v) pullulan in 0.2 M phosphate buffer (pH 4.0) and 250 μL enzyme solution in 0.2 M phosphate buffer (pH 4.0), was incubated at 60°C for 20 min. Then, the release of aldehyde groups was assayed by the dinitrosalicylic acid (DNS) method by measuring the absorbance at 540 nm spectrophotometrically. One unit of pullulanase was defined as the amount of the enzyme that resulted in release 1 μmol of aldehyde groups per minute at 60°C. Organophosphorus hydrolase activity was assayed as described previously [6]. One unit of OPHC2 activity was defined as the amount of the enzyme that liberated 1 μmol of *p*-nitrophenol per minute at 37°C.

In addition, both *B. subtilis* WB600 and *B. subtilis* WB600 harboring different fusion plasmids were cultivated in 50 mL SB medium[7]. After culturing for 12 h, samples were taken for determination of pullulanase and OPHC2 activities every 3 hours until 39 hours. The method as the same as the mentioned above.

Table S1. Strains and Plasmids used in this study

**Materials**	**Relevant characteristics**	**sources**
E. coli Trans1-T1	$F^{-}\Phi80$(*lac*Z)ΔM15Δ*lac*X74*hsd*R(r_k^-,m_k^+)Δ*rec*A 1398*end*A1*ton*A	This study
B. subtilis WB600	*apr, nprA, epr, bpf, mpr, nprB, trpC2*	This study
pUBC19	*E. coli-B. subtilis* shuttle vector	[8]
pDK	*bgaB* gene donor	[1]
P-free-bgaB-pUBC19	pUBC19 carrying *bgaB* gene	This study
P43-bgaB-pUBC19	P-free-bgaB-pUBC19 inserted P43 Promoter	This study
P*ydc*-bgaB-pUBC19	P-free-bgaB-pUBC19 inserted P*ydc* Promoter	This study
P*yddA*-bgaB-pUBC19	P-free-bgaB-pUBC19 inserted P*yddA* Promoter	This study
P*msm*-bgaB-pUBC19	P-free-bgaB-pUBC19 inserted P*msm* Promoter	This study
P*pbp*-bgaB-pUBC19	P-free-bgaB-pUBC19 inserted P*pbp* Promoter	This study

P*yvl*-bgaB-pUBC19	P-free-bgaB-pUBC19 inserted P*yvl* Promoter	This study
P*ylb*-bgaB-pUBC19	P-free-bgaB-pUBC19 inserted P*ylb* Promoter	This study
P*yob*-bgaB-pUBC19	P-free-bgaB-pUBC19 inserted P*yob* Promoter	This study
P*yddF*-bgaB-pUBC19	P-free-bgaB-pUBC19 inserted P*yddF* Promoter	This study
F1-bgaB-pUBC19	P-free-bgaB-pUBC19 inserted the fragment -381～+43 of P*ylb* promoter	This study
F2-bgaB-pUBC19	P-free-bgaB-pUBC19 inserted the fragment -233～+43 of P*ylb* promoter	This study
F3-bgaB-pUBC19	P-free-bgaB-pUBC19 inserted the fragment -154～+43 of P*ylb* promoter	This study
F4-bgaB-pUBC19	P-free-bgaB-pUBC19 inserted the fragment -78～+43 of P*ylb* promoter	This study
F5-bgaB-pUBC19	P-free-bgaB-pUBC19 inserted the fragment -21～+43 of P*ylb* promoter	This study
E-R-pUBC19	pUBC19 harbouring *egfp* and *mApple*	This study
P*ylb*-R-P43-G-pUBC19	*mApple* directed by P*ylb* and *egfp* directed by P43 in E-R-pUBC19 vector	This study
P*ylb*-G-P43-R-pUBC19	*egfp* directed by P*ylb* and *mApple* directed by P43 in E-R-pUBC19 vector	This study
F4-egfp-pUC19	*egfp* directed by F4 in pUC19 vector	This study
F4-G-P43-R-pUBC19	*egfp* directed by F4 and *mApple* directed by P43 in E-R-pUBC19 vector	This study
pM4843	Mutagenesis from bp -48 to -43 of F4 in F4-G-P43-R-pUBC19 vector	This study
pM4338	Mutagenesis from bp -43 to -38 of F4 in F4-G-P43-R-pUBC19 vector	This study
pM3833	Mutagenesis from bp -38 to -33 of F4 in F4-G-P43-R-pUBC19 vector	This study
pM3328	Mutagenesis from bp -33 to -28 of F4 in F4-G-	This study

	P43-R-pUBC19 vector	
pM2823	Mutagenesis from bp -28 to -23 of F4 in F4-G-P43-R-pUBC19 vector	This study
pM2318	Mutagenesis from bp -23 to -18 of F4 in F4-G-P43-R-pUBC19 vector	This study
pM1813	Mutagenesis from bp -18 to -13 of F4 in F4-G-P43-R-pUBC19 vector	This study
pM1308	Mutagenesis from bp -13 to -8 of F4 in F4-G-P43-R-pUBC19 vector	This study
pM0803	Mutagenesis from bp -8 to -3 of F4 in F4-G-P43-R-pUBC19 vector	This study
pM0301	Mutagenesis from bp -3 to -1 of F4 in F4-G-P43-R-pUBC19 vector	This study
P*ylb*-ZDs-Pul-pUBC19	pUBC19 inserted promoter F3, the signal peptide sequence of the amylase and pullulanase gene	This study
P43-ZDs-Pul-pUBC19	pUBC19 inserted promoter P43, the signal peptide sequence of the amylase and pullulanase gene	This study
P*ylb*-ZDs-OPHC2-pUBC19	pUBC19 inserted promoter F3, the signal peptide sequence of the amylase and organophosphorus hydrolase gene	This study
P43-ZDs-OPHC2-pUBC19	pUBC19 inserted promoter P43, the signal peptide sequence of the amylase and organophosphorus hydrolase gene	This study

Table S2. Primers and oligonucleotides used in this study.

Primers	Sequences(5′→ 3′)	Restriction sites
Primers for real-time PCR		
argD-up	GGACACTGCCATGAAGCGGTGAC	

argD-down	TTCTGGCAAGCTTTATCGCACCT
hut-up	ACTGCATAAAGAGCGTCGGATCGGC
hut-down	TGGAAGCGGTTTCAATTGCGGCTAC
yceC-up	CGGTCCGAATATCGACTGTGACGCTT
yceC-down	AATTTGCTCGTCATCGCCCGCACCGT
ydcO-up	TGATGTTGAAGCTTTAAAGGCTG
ydcO-down	GGACGAGTTGACCAGATACACCA
yddA –up	AGCTTAGGTTTTAATGTGGGCATTCCCT
yddA -down	GCTAGTCATTCGACCGTCTGGCAAGTAT
yuaF-up	TTCAGGGCTGTCAGAAGGCATTCCG
yuaF-down	GTGAAGCAATACCACCAGCATGATCG
arsB-up	GCCAGATAAACCGGAGTATATGA
arsB-down	ACCATTGCGGAATAACTGTTACA
carA-up	TTGAAAGCAAGAAACCGCAAGTA
carA-down	TAGAGGCTGTGACTGTCGCACCC
msmX-up	CTTTAACCTTCATATTGCCGATA
msmX-down	CCCTGTCCTTTGGCGCTACATCA
pbpE-up	TTTGGCTATGCGGAAATGACGGA
pbpE-down	TTCAATAAATGCCGAATCGTTAC
rapA-up	CTGAAGCCGAACGGGTCAAGCTC
rapA-down	AAACTCTAGCTGCGACGTGTCCT
sacV-up	AAGGAGAGTTTTTAACTGCGAGA
sacV-down	TGGGCCTTTGTAACAATAACACT
sigW-up	TGCATGAGGCGGAGGATATTGCACA
sigW-down	ACATGGTCAAGCCTTCCGTACCAGC
yvlA-up	TCAGTGTGTTCATTGCGCCAATCATTGT
yvlA-down	GCAAGCTGTCTCCATCAATCGCAAC
yxbB-up	GGCTGAAGAATGCAGAACGTACA
yxbB-down	GGGTTAATATCGACAGCATGCAC

ylbP-up	TGGCGATAAACTTGTAGCACGAA	
ylbP-down	ATTCAACCAAGGCTCTCCCGTAT	
yobJ-up	AGTTGCATTGCTATTCAATCAAAGACAA	
yobJ-down	TGATAGCTTCCCCTTTTTCCCACTATATTCT	
yqeZ-up	ATGGCCGATTTGATAACCGAAAG	
yqeZ-down	TCTGCCAGCCAAAGTGATTGAGC	
yxjJ-up	TGAATACTTTTTGGCATTTTATCCG	
yxjJ-down	CTGCTGGCAGAGGGCTTGTCTTTTG	
argG-up	CAGGCCCACACAATGTATGA	
argG-down	GGCGCGATGACTTCTAGGTC	
yddF-up	GGAAATCACTAACAATGAGACTGTG	
yddF-down	ATGACCACTTCCAAGAATCC	
cdd-up	GCATATGCGCCGTATTCCAA	
cdd-down	CCAGGGGTGTCAGCCGCAAC	
16srRNA-up	AAGTCCCGCAACGAGCGCAA	
16srRNA-down	TCGCGGTTTCGCTGCCCTTT	
Primers for recombinant plasmids		
bgaB-up	ATA**CTGCAGCTCGAG**ATGAATGTGTTATCCTCAA	*Pst* I *Xho* I
bgaB-down	ATA**GGATCC**GCATATTATGTTGCCAACT	*Bam* HI
P43-up	ATA**CTGCAG**TGATAGGTGGTATGTTTTCG	*Pst* I
P43-down	ATA**CTCGAG**GTGTACATTCCTCTCTTACCTATAA	*Xho* I
P*ydc*-up	ATA**CTGCAG**ATGCATAATGTCAATCTGTTGAA	*Pst* I
P*ydc*-down	ATA**CTCGAG**CATGCAATCTCTGCTTAAACTTC	*Xho* I
P*yddA*-up	ATA**CTGCAG**GAGTGAATGCAACAATCATTACTG	*Pst* I
P*yddA*-down	ATA**CTCGAG**CATTTTTCATATCCCCATTTCATT	*Xho* I
P*msm*-up	ATA**CTGCAG**AGTGTCTGCGAAAACATTAC	*Pst* I
P*msm*-down	ATA**CTCGAG**CTAACATCCCCCTTTGTTAT	*Xho* I
P*pbp*-up	ATA**CTGCAG**AGATGGCAAGTTAGTTACGC	*Pst* I
P*pbp*-down	ATA**CTCGAG**TCCTCCACCTCCCATATCTC	*Xho* I

P*rap*-up	ATA**CTGCAG**GTACGACAAACTATCCCGAAGA	*Pst* I
P*rap*-down	ATA**CTCGAG**CGTCTGCTTCATCCTCAATTAA	*Xho* I
P*sig*-up	ATA**CTGCAG**AATCCCGTACGGGTCACCAT	*Pst* I
P*sig*-down	ATA**CTCGAG**AATCATCATTTCCATATTTATCTAACC	*Xho* I
P*yvl*-up	ATA**CTGCAG**TTCAAAACAAAAAAGGCAAGAT	*Pst* I
P*yvl*-down	ATA**CTCGAG**TTCATTCCACACTCCTATTG	*Xho* I
P*ylb*-up	ATA**CTGCAG**CATCGTCGAACGCGCTCCAT	*Pst* I
P*ylb*-down	ATA**CTCGAG**ACGTTCTACCTTTGTCAAACAA	*Xho* I
P*yob*-up	ATA**CTGCAG**ATTCGGCGTTTTGGTTTTAGGC	*Pst* I
P*yob*-down	ATA**CTCGAG**TTAAGCTTCTCCCCTTCTCT	*Xho* I
P*yqe*-up	ATA**CTGCAG**CTTCTTGAGCGTCTCAACCA	*Pst* I
P*yqe*-down	ATA**CTCGAG**TCCTTTGATTTGAAGCAAGG	*Xho* I
P*yddF*-up	ATA**CTGCAG**TGTGCGAGTAGATCTAGGTACT	*Pst* I
P*yddF*-down	ATA**CTCGAG**AGTGATTTCCTTTTTCATATTATCCC	*Xho* I
P43-G-up	ATA**TCTAGA**TGATAGGTGGTATGTTTTCG	*Xba* I
P43-G-down	ATA**GAGCTC**GTGTACATTCCTCTCTTACCTATAA	*Sac* I
P43-R-up	ATA**CCTAGG**TGATAGGTGGTATGTTTTCG	*Avr* II
P43-R-down	ATA**CTCGAG**GTGTACATTCCTCTCTTACCTATAA	*Xho* I
P*ylb*-G-up	ATA**TCTAGA**CATCGTCGAACGCGCTCCAT	*Xba* I
P*ylb*-G-down	ATA**GAGCTC**ACGTTCTACCTTTGTCAAACAA	*Sac* I
P*ylb*-R-up	ATA**CCTAGG**CATCGTCGAACGCGCTCCAT	*Avr* II
P*ylb*-R-down	ATA**CTCGAG**ACGTTCTACCTTTGTCAAACAA	*Xho* I
F1-up	ATA**CTGCAG**AAAAAACAGATTGAAGCTGA	*Pst* I
F2-up	ATA**CTGCAG**TTCTTGCCGCCTCGTCACAA	*Pst* I
F3-up	ATA**CTGCAG**CCGATAATTCCAATTTTCAT	*Pst* I
F4-up	ATA**CTGCAG**ACTTCTCAAAGATCCCATGT	*Pst* I
F5-up	ATA**CTGCAG**TAAAGCGTTTACAATATATGTAG	*Pst* I
PB1	AATCCCTAAAGATACTAAATTC	
PB2-up	CCTTTTAGTCCAGCTGATTT	

PB2-down	TTCCTCTGGCCATTGCTCTG	
PB3-up	ATGTAAATCGCTCCTTTTTAGG	
PB3-down	CTCCGTAACAAATTGAGGATAA	
F4-egfp-up	ATA**TCTAGA**ACTTCTCAAAGATCCCATGT	*Xba* I
F4-egfp-down	ATA**AAGCTT**ACTGAGCAAAAAAAATCCTG	*Hind* III
M4843-up	TCCCATGTGCTTAAAATT***CCCTGG***TAAATATTTGGATTTTTT	
M4338-up	TGTGCTTAAAATTAAAGT***GGCCCG***ATTTGGATTTTTTAAATA	
M3833-up	TTAAAATTAAAGTTTAAA***GCGGGT***GATTTTTTAAATAAAGCG	
M3328-up	ATTAAAGTTTAAATATTT***TTCGGG***TTTAAATAAAGCGTTTAC	
M2823-up	AGTTTAAATATTTGGATT***GGGGCC***ATAAAGCGTTTACAATAT	
M2318-up	AAATATTTGGATTTTTTA***CCGCCC***GCGTTTACAATATATGTA	
M1813-up	TTTGGATTTTTTAAATAA***CTATGG***TACAATATATGTAGAAAC	
M1308-up	ATTTTTTAAATAAAGCGT***GGCACC***TATATGTAGAAACAACAA	
M0803-up	TTAAATAAAGCGTTTACA***CGCGCG***GTAGAAACAACAAAGGGG	
M0301-up	TAAAGCGTTTACAATATA***GTG***AGAAACAACAAAGGGGGA	
M-down	TCTTCACCTTTGGACACCATGAGCTC	
P*ylb*(F4)-up	ATA**TCTAGA**CCGATAATTCCAATTTTCAT	*Xba* I
P*ylb*-down	GTTTCCTCTCCCTCTCATACGTTCTACCTTTGTCAA	
ZDsylb-up	TTGACAAAGGTAGAACGTATGAGAGGGAGAGGAAAC	

ZDs-down	ATA<u>**CTCGAG**</u>GGCTGATGTTTTTGTAATCG	*Xho* I
P43-up	ATA<u>**TCTAGA**</u>TGATAGGTGGTATGTTTTCG	*Xba* I
P43-down	GTTTCCTCTCCCTCTCATGTGTACATTCCTCTCTTA	
ZDs43-up	TAAGAGAGGAATGTACACATGAGAGGGAGAGGAAAC	
Pul-up	ATA<u>**CTCGAG**</u>GCCGCACCGGCACAACAGA	*Xho* I
Pul- down	ATA<u>**CTGCAG**</u>TCAGCGGTCGCTACGGATC	*Pst* I
OPHC2-up	ATA<u>**CTCGAG**</u>CCTGCTGTAAGTAACGCTTATT	*Xho* I
OPHC2-down	ATA<u>**CTGCAG**</u>CTATTTACCATCAGATGGGCTT	*Pst* I

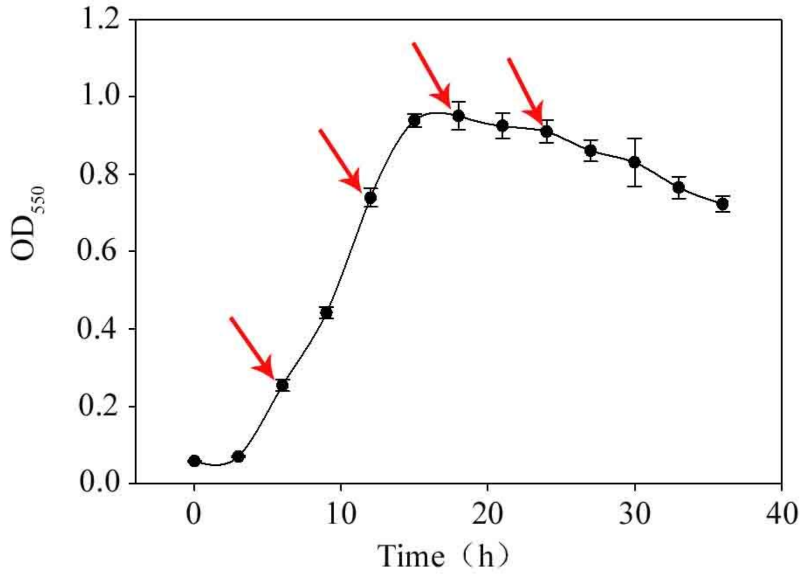

Figure S1. Growth curve of *B. subtilis* WB600 in LB medium. *B. subtilis* WB600 was grown in LB medium containing 5 μg/mL tetracycline. The y-axis presents the average optical densities at 550 nm of bacterial culture at each time point. Data are averages of three independent experiments. Cells were collected for RNA extraction after 6, 12, 18, and 24 h of culture, and it is indicated by the red arrow.

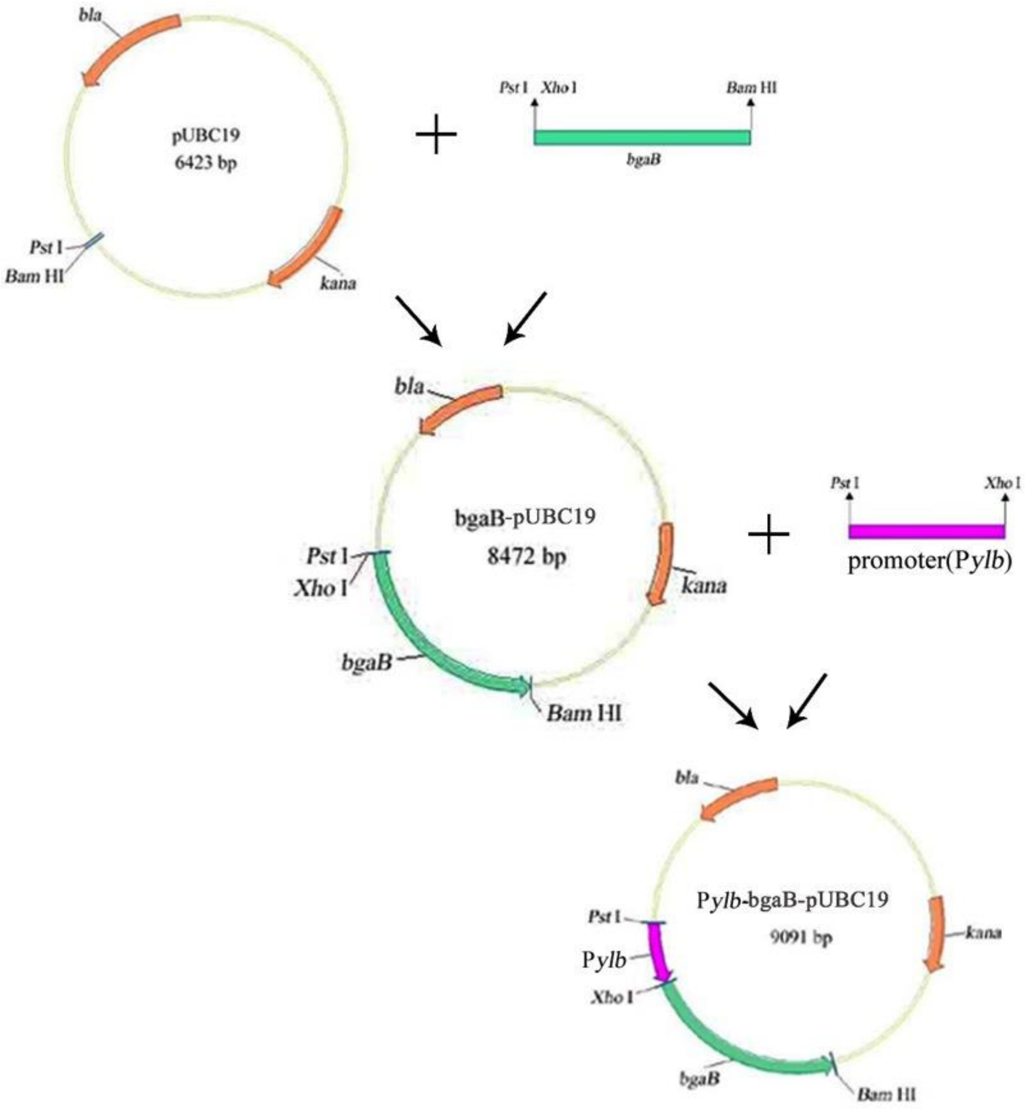

Figure S2. Construction of the fusion plasmid Promoter-bgaB-pUBC19.

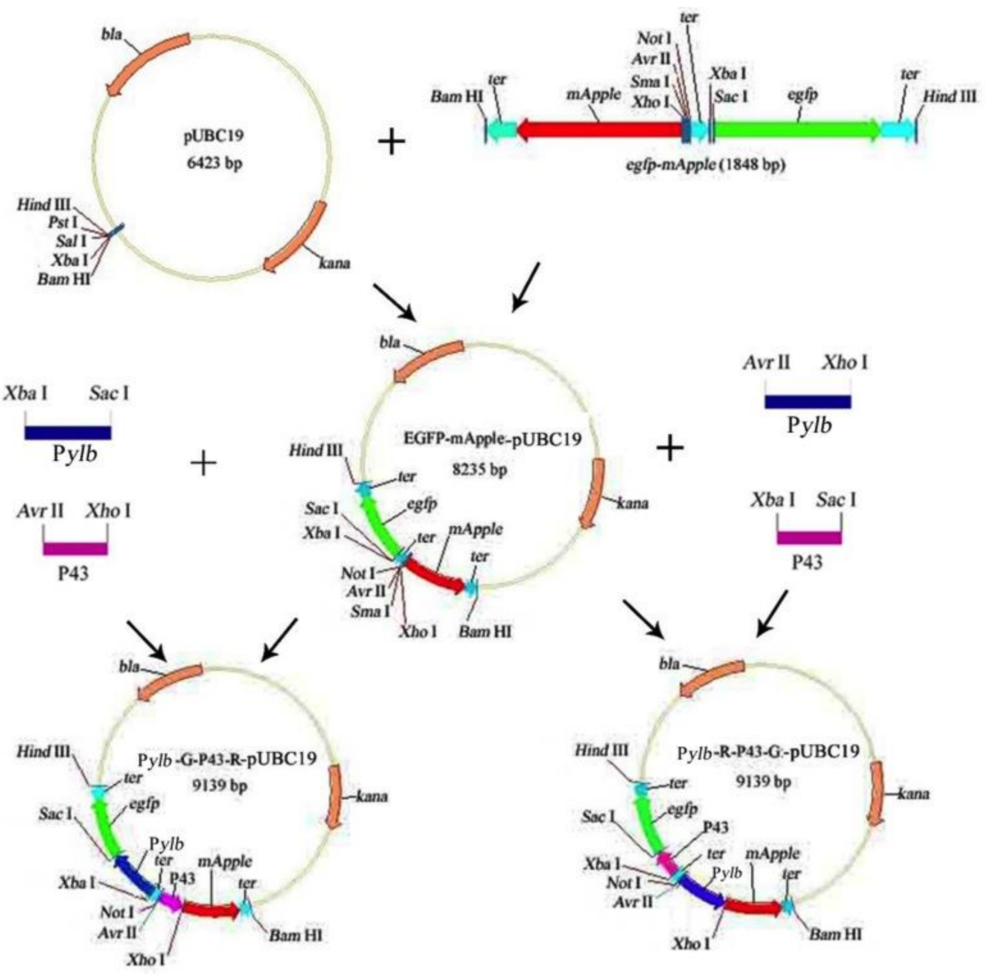

Figure S3. Construction of the fusion plasmids P*ylb*-G-P43-R-pUBC19 and P*ylb*-R-P43-G-pUBC19, which contain two promoters and two genes encoding fluorescent proteins.

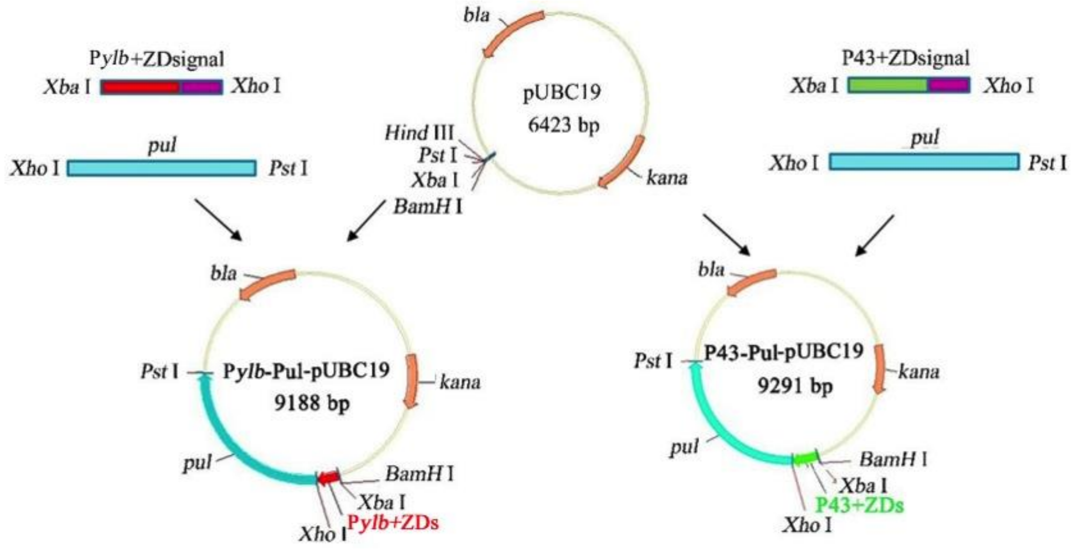

Figure S4. Construction of the fusion plasmids P*ylb*-ZDs-Pul-pUBC19 and P43-ZDs-Pul-pUBC19.

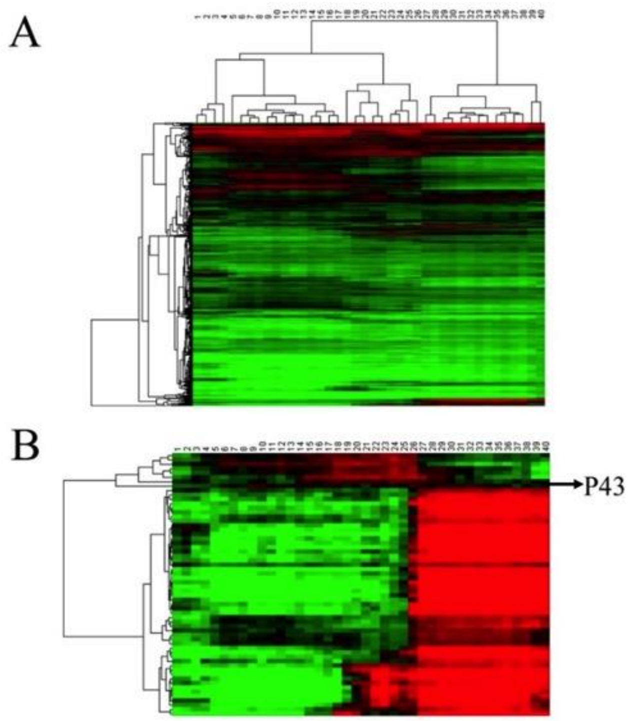

Figure S5. Gene expression profiles over time during batch culture of *B. subtilis*.

(A) A total of 4,169 genes was subjected to SAM analysis. Each column represents a time point (total of 40 time points), and each row represents a gene (total of 4169 genes). The greater the number of red dots, the higher the gene expression level. (B) Fifty-eight genes expressed at a high level were subjected to SAM analysis. Each column represents a time point (total of 40 time points), and each row represents a gene (total of 59 genes). The arrowhead indicates the P43 control gene. The greater the number of red dots, the higher the gene expression level.

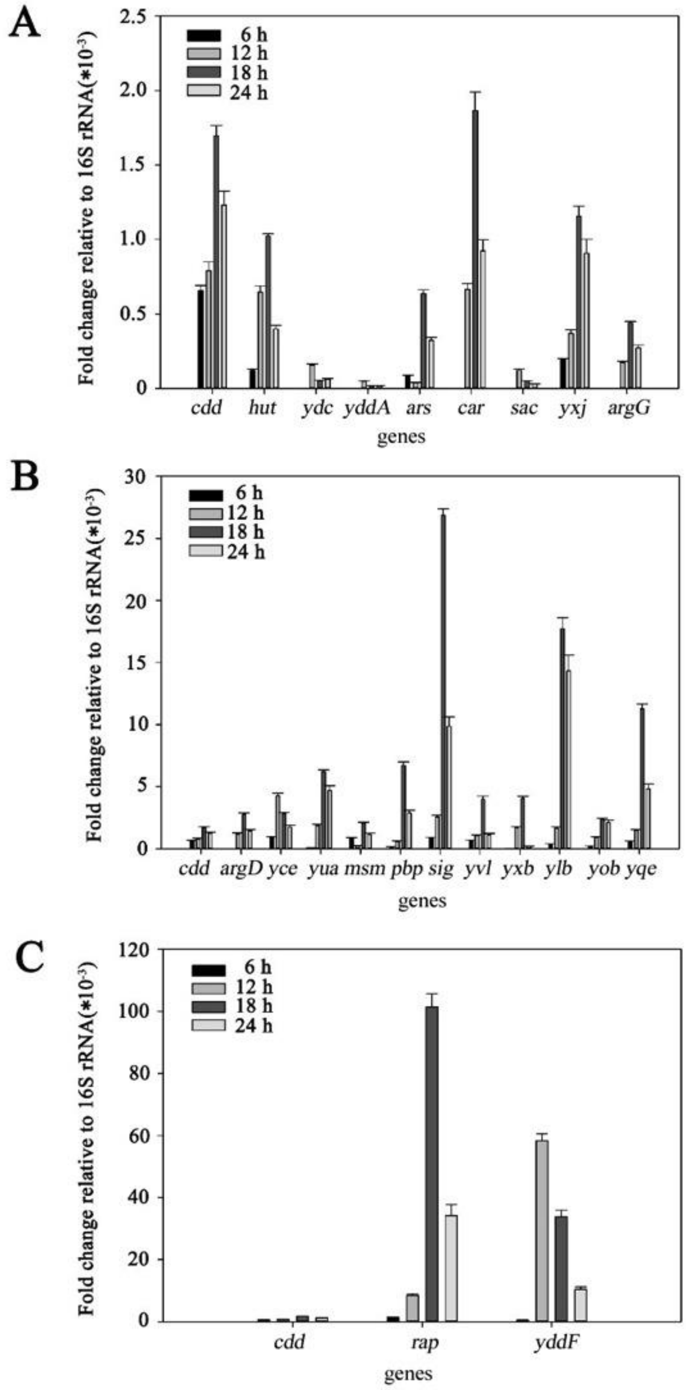

Figure S6. Comparative analysis of gene transcript levels in *B. subtilis.* The x-axis shows high-level expressed genes of gene expression profiles. The y-axis shows the fold-change in the transcript level of the selected genes relative to that of the reference gene, 16S rRNA. Comparative analysis of the transcript levels of the control genes of (A) genes of lower transcript levels than *cdd* (controlled by P43); (B) genes of higher transcript levels than *cdd*; and (C) genes of the highest transcript levels. Data are averages of three independent experiments.

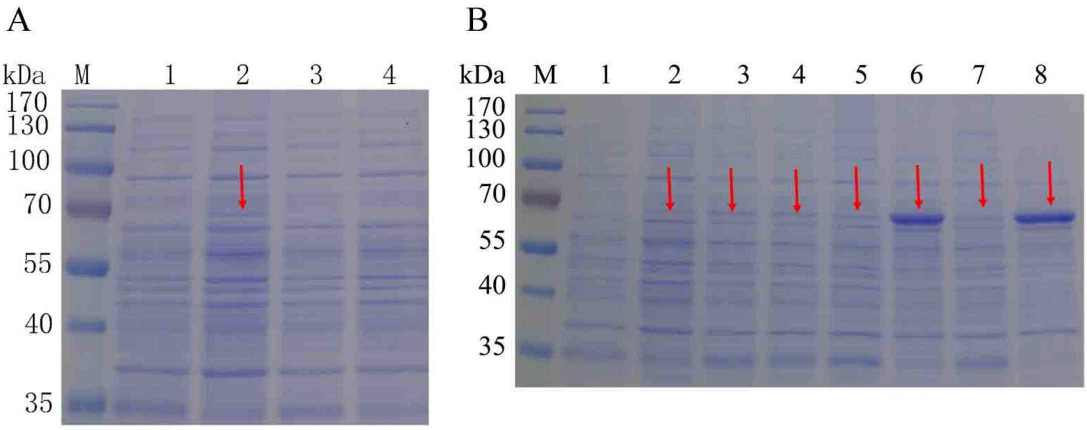

Figure S7. SDS-PAGE analysis of BgaB in crude *B. subtilis* extract which containing recombinant plasmid promoter-bgaB-pUBC19. (A) M: protein molecular mass marker; 1: P-free as a negative control. 2: P43 as a positive control. 3 and 4: P*ydc* and P*yddA*, respectively. (B) M: protein molecular mass marker; 1: P-free as a negative control. 2: P43 as a positive control. 3, 4, 5, 6, 7 and 8: P*msm*, P*pbp*, P*yvl*, P*ylb*, P*yob*, and P*yddF*, respectively, and the red arrows indicate target protein BgaB.

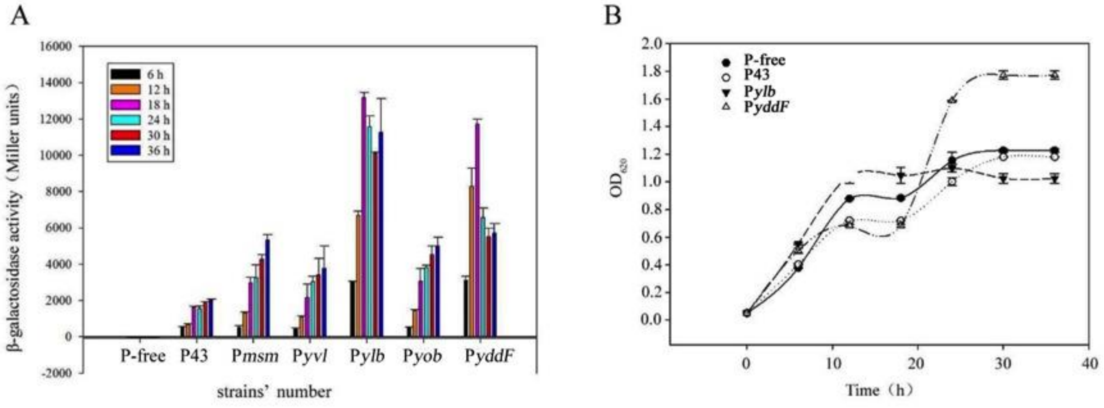

Figure S8. β-Galactosidase activities and growth curve of the candidate promoter clones in *B. subtilis* according to growth stage. Data are averages of three independent experiments.

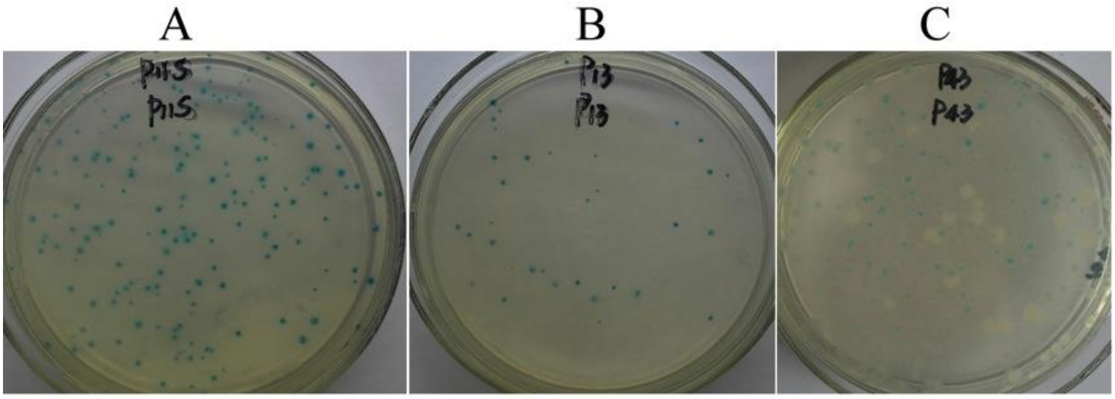

Figure S9. The observation of the P*rap*, P*sig* and P43 promoters clones in *B. subtilis* on the LB agar containing 10 μg/mL kanamycin with x-gal. A: P*rap* promoter clones in *B. subtilis*; B: P*sig* promoter clones in *B. subtilis*; C: P43 promoters clones in *B. subtilis*.

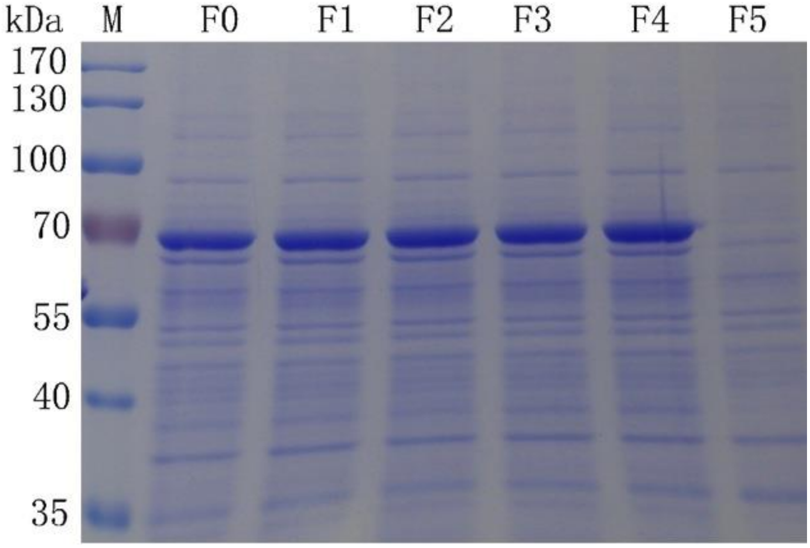

Figure S10. SDS-PAGE analysis of BgaB in crude extracts of various *B. subtilis* strains. M: protein molecular mass marker; 1: crude extract of the *B. subtilis* strain harboring the wild-type promoter P*ylb* (F0) harvested after culture for 18 h. 2, 3, 4, 5, and 6: crude extracts of *B. subtilis* strains, in which the promoters had been deleted (F1, F2, F3, F4, and F5, respectively), harvested after culture for 18 h.

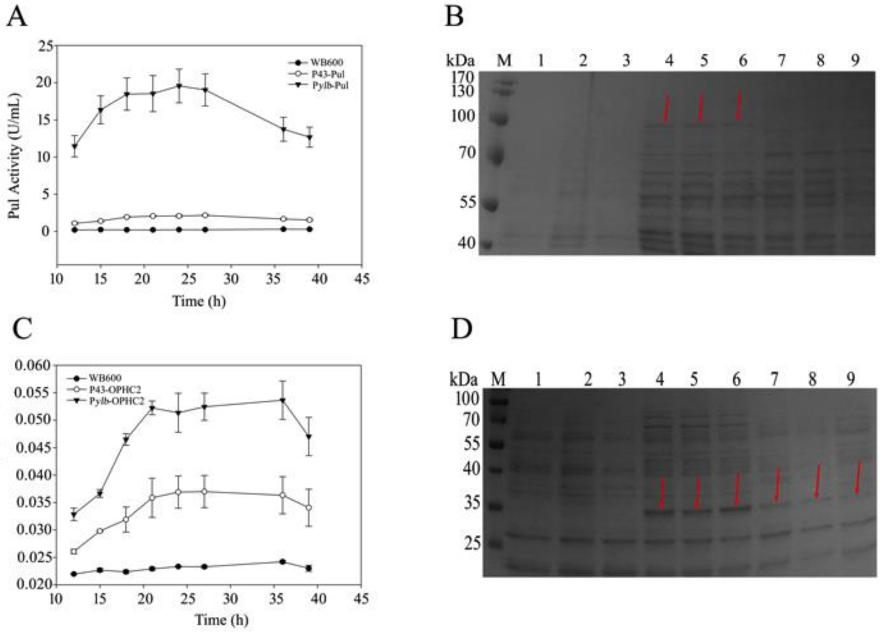

Figure S11. Pullulanse and OPHC2 activities of *B. subtilis* strains harboring various promoters in SB medium within 39 hours. (A) Production of pullulanse by *B. subtilis* WB600 harboring the recombinant plasmid P*ylb*-ZDs-Pul-pUBC19 (indicated by P*ylb*-Pul), P43-ZDs-Pul-pUBC19 (P43-Pul), or no recombinant plasmid (WB600) within 39 hours. (B) SDS-PAGE analysis of pullulanse activity in the culture supernatant after 24 h of culture. M: protein molecular mass marker. 1-3: WB600-1, WB600-2 and WB600-3 (as a negative control). 4-6: P*ylb*-Pul-1, P*ylb*-Pul-2 and P*ylb*-Pul-3. 7-9: P43-Pul-1, P43-Pul-2 and P43-Pul-3, and the arrows indicate target protein pullulanse. (C) Production of OPHC2 by *B. subtilis* WB600 harboring the recombinant plasmid P*ylb*-ZDs-OPHC2-pUBC19 (indicated by P*ylb*-OPHC2), P43-ZDs-OPHC2-pUBC19 (P43- OPHC2), and no recombinant plasmid (WB600) within 39 hours. (D) SDS-PAGE analysis of OPHC2 in the culture supernatant after 24 h of culture. M: protein molecular mass marker. 1-3: WB600-1, WB600-2 and WB600-3. 4-6: P*ylb*-OPHC2-1, P*ylb*-OPHC2-2 and P*ylb*-OPHC2-3. 7-9: P43-OPHC2-1, P43-OPHC2-2 and P43-OPHC2-3, and the arrows indicate target protein OPHC2. Data are averages of three independent experiments.

References:

1 Yuan, G. & Wong, S. L. Regulation of groE expression in *Bacillus subtilis*: the involvement of the sigma A-like promoter and the roles of the inverted repeat sequence (CIRCE). *J. Bacteriol.* **177**, 5427-5433 (1995).

2 Hirata, H., Fukazawa, T., Negoro, S. & Okada, H. Structure of a beta-galactosidase gene of *Bacillus stearothermophilus*. *J. Bacteriol.* **166**, 722-727 (1986).

3 JH, M. Experiments in Molecular Genetics. *Cold Spring Harbor Laboratory* (1972).

4 Riggs, PaulÂ D. *et al.* High-Level Expression of *Bacillus naganoensis* Pullulanase from Recombinant *Escherichia coli* with Auto-Induction: Effect of lac Operator. *PLoS One* **8**, e78416, (2013).

5 Chen, W.-B., Nie, Y. & Xu, Y. Signal Peptide-Independent Secretory Expression and Characterization of Pullulanase from a Newly Isolated Klebsiella variicola SHN-1 in *Escherichia coli*. *Appl. Biochem. Biotech.* **169**, 41-54, (2012).

6 Chu, X.-Y. *et al.* Expression of organophosphorus hydrolase OPHC2 in *Pichia pastoris*: Purification and characterization. *Protein Expres. Purif.* **49**, 9-14, (2006).

7 Durban, M. A., Silbersack, J., Schweder, T., Schauer, F. & Bornscheuer, U. T. High level expression of a recombinant phospholipase C from *Bacillus cereus* in *Bacillus subtilis*. *Appl. Microbiol. Biotechnol.* **74**, 634-639, (2007).

8 Zhang, X.-Z., Cui, Z.-L., Hong, Q. & Li, S.-P. High-level expression and secretion of methyl parathion hydrolase in *Bacillus subtilis* WB800. *Appl. Environ. Microbiol.* **71**, 4101-4103 (2005).
